# Hydrogel containing minocycline and zinc oxide-loaded serum albumin nanopartical for periodontitis application: preparation, characterization and evaluation

**DOI:** 10.1080/10717544.2019.1571121

**Published:** 2019-03-01

**Authors:** Jie Mou, Zongxiang Liu, Jie Liu, Jianwu Lu, Wentao Zhu, Dongsheng Pei

**Affiliations:** a Jiangsu Key Laboratory of New drug and Clinical Pharmacy, Xuzhou Medical University, Xuzhou, China;; b School of Pharmacy, Xuzhou Medical University, Xuzhou, China;; c Affiliated Stomatological Hospital of Xuzhou Medical University, Xuzhou, China;; d Department of pathology, Xuzhou Medical University, Xuzhou, China

**Keywords:** Minocycline, zinc oxide, serum albumin, nano-hydrogel, periodontitis

## Abstract

Periodontal disease is a complex problem which often interrelates with several serious systemic diseases. However, the satisfactory clinical therapy has yet to be achieved. Herein, serum albumin microspheres containing minocycline and zinc oxide nanoparticals (ZnO NPs) were prepared and incorporated in a Carbopol 940^®^ hydrogel. Compared with 2% minocycline ointment (Perio^®^), the hydrogel has shown obvious therapy effects and the ability of gingival tissue self-repairing. The serum albumin microspheres containing 0.06% of minocycline and 0.025% of ZnO NPs presented an average size of 139 ± 0.42 nm using electrophoretic light scattering (*n* = 3). Photomicrographs obtained by TEM showed homogeneous and spherical-shaped particles. The encapsulation efficiency was 99.99% for minocycline and the slow-release time was more than 72 h with pH-sensitive property. The *in vitro* skin adhesion experiment showed that the largest bioadhesive force is 0.35 N. Moreover, the hydrogel showed broad-spectrum antimicrobial and effective antibacterial ability when concentration of the ZnO NPs was over 0.2 µg/mL. The cell survival rates were more than 85% below 0.8 mg/L of ZnO NPs, which proved its low toxicity and high security.

## 1. Introduction

Periodontitis, one of the common periodontal disease, is an infectious disease caused by biofilms with a mixed microbial etiology and leads to progressive destruction of the teeth-supporting tissues (Hajishengallis, 2015). Except for the major cause of tooth loss, periodontitis has also an association with variety of diseases such as atherosclerotic vascular disease, diabetes mellitus, and chronic nephritis (Teratani et al., 2013; Holmlund et al., 2017; Graziani et al., 2018; Liljestrand et al., 2018).

The universal clinical treatment of periodontitis is the scaling and root planning (SRP) to remove the supra- and subgingival biofilm from the root surface in order to eliminate the pathogenic bacteria (Smiley et al., 2015; Ralee et al., 2016). However, SRP is highly depending on the skills of the clinician and limited in the inability to reach into the bacteria residing deep in the periodontal pocket or the furcation areas. Furthermore, infections resulting from microbial biofilm formation remain a serious challenge to patients.

Herein, we designed an albumin-based drug delivery system and incorporated into carbopol hydrogel networks, in which zinc oxide (ZnO) was introduced to increase the stability of albumin nanoparticles and provide coordination sites to load antibacterial drug. The hydrogel can realize pH-responsive to release the antibacterial drug. The antibacterial properties were investigated by the effect evaluation of ZnO nanoparticals according to the different sizes and contents of ZnO.

## 2. Materials and methods

### 2.1. Materials

Znic oxide nanoparticals (ZnO NPs) were synthesized in our laboratory. Minocycline, analytical grade, was purchased from Sigma (Sigma-Aldrich, USA) and kept away from light. Albumin and carbopol 940 was obtained from Jiangsu Chemical Company (China). Minocycline ointment (Perio^®^) was supplied by the affiliated stomatologica hospital of Xuzhou Medical University（Xuzhou, China).

For cell culture studies, cell culture medium (PBS), 0.25% trypsin-EDTA, and fetal bovine serum (FBS) were obtained from Gibco (Grand Island, NE) and Cell Counting Kit-8 (CCK-8) were purchased from Sigma and stored at −4 °C in the dark.

The strains were obtained from Institute of Biochemistry and Cell Biology, Institute for Biological Sciences, Chinese Academy of Science (Shanghai, China). Water used in this study was doubly-distilled and deionized. All other chemicals and solvents were analytical grade.

SD rats were purchased from the animal center of Xuzhou medical university. All animal care and the experimental protocols were approved by the Animal Ethics Committee in Xuzhou Medical University.

### 2.2. Methods

#### 2.2.1. Synthesis and characterization of ZnO NPs

ZnO NPs were synthesized using the modified reversed-phase microemulsion method which utilized zinc acetate as precursor (Mou et al., 2017). Briefly, hexadecyl trimethyl ammonium bromide (CTAB, 0.3644 g, 1 mmol) was dissolved in cyclohexane (10 mL). The suspension was heated at 80 °C for 1 h and cooled to room temperature under stirring overnight. Next, triethanolamine (5 mL) was added dropwise under vigorous stirring until the milk-white emulsion was formed. Zinc acetate (Zn(CH_3_COO)_2_ · 2H_2_O) (2.19 g, 1 mmol) was dissolved in distilled water (100 mL) whilst stirring to obtain the 1 mol/L zinc solution. Zinc acetate solution (0.2 mL, 1 mol/L) was injected into the abovementioned emulsion slowly under moderate stirring until the mixture turn cleared and stirred for another 1 h. Then the solution was sonicated at 200 W until the white nanocrystalline formed. After washing, the powder was allowed to dry in an oven at 400 °C for 1 h. Synthesized ZnO NPs were characterized for the particle size and zeta potential by NICOMP 380ZLS zeta potential/particle size analyzer (PSS, USA). X-ray powder diffractometry (Bruker AXS D8 Advance Diffractometer), morphology (JSM-6510, JEOL, Japan) and transmission electron microscope (TEM, JEM 1230, JEOL) operating at 100 kV.

#### 2.2.2. Preparation and characterization of Mino-ZnO@Alb NPs

The albumin microspheres were prepared through the emulsification cross-linking approach. The formulation was prepared by dissolving ZnO NPs (1 mg), albumin (1 mg), and minocycline (1 mg) in PBS solution (pH 8.0) in a vial. All ingredients were kept for 10 min at 40 °C under magnetic stirring at 450 rpm. Ethanol (4 mL) and glutaraldehyde (20 μL) was added successively under magnetic stirring at 600 rpm for 30 min. Finally, the albumin microspheres were scattered under sonication for 30 s. The drug-loaded nanopartical was denominated Mino-ZnO@Alb NPs. Placebo nanoparticals (Alb NPs), without ZnO nanopartical and minocycline, were prepared for comparison. The average diameter and Zeta potential were determined by dynamic light scattering (DLS) with Zetasizer (Nano-ZS90, Malvern Instrument, Malvern, England) at room temperature. Measurements were conducted in triplicate at 25 °C for all samples appropriately diluted in distilled water. The concentration of minocycline was analyzed with ultraviolet absorption at their maximum wavelength (236 nm) on a UV spectrophotometer.

#### 2.2.3. Preparation of hydrogels containing Mino-ZnO@Alb NPs

Drug-loaded hydrophilic gels (Mino-ZnO@Alb NPs HG) were prepared by dispersing 1% (*w*/*w*) of Carbopol 940^®^ (polymer of acrylic acid) in the nanoparticle dispersion (Mino-ZnO@Alb NPs). After 24 h, the dispersion was neutralized with triethanolamine. Diazolidinyl urea (0.3%, *w*/*w*) was added as a preservative. Hydrogels bases (B-HG) were prepared using distilled water instead of the nanopartical suspension.

#### 2.2.4. Encapsulation efficiency (EE) and drug-loading content (LD) assay of Mino-ZnO@Alb NPs

EE was determined for separating free drug from Mino-ZnO@Alb NPs using ultrafiltration centrifuge tubes (Millipore Corporation, MA). Drug content in the nanoparticles (LD) was measured using a dissolution method.

In brief, 1 mL Mino-ZnO@Alb NPs were added in ultrafiltration tubes, then centrifuged at 3000 rpm for 10 min to separate free minocycline from preparations. One hundred microliters of filtrate was demulsified with methanol and then metered to a volume of 10 mL. The concentration of minocycline was determined by UV spectrophotometer using linearly regressed standard curve at 236 nm. EE and LD values were calculated by the following equations:

$$\text{EE}\%=(W_{t}-W_{f})/W_{t}\times 100\%$$

$$\text{LD}\%=(\text{EE}\times W_{t})/W_{\text{mino-ZnO}@\text{Alb}\text{NPs}}\times 100\%$$

Where *W*f is the amount of free minocycline not entrapped in the Alb nanoparticles, *W*t is the total amount of Minocycline in the Alb nanoparticle delivery systems and *W*mino-ZnO@Alb NPs is the total weight of Mino-ZnO@Alb NPs. Each experiment was carried out in triplicate.

#### 2.2.5. Antibacterial ability of Mino-ZnO@Alb NPs

The inhibition zone method was used to evaluate the antibacterial activity of the antibacterial delivery system. Fresh culture of *S. oralis/S. mitis*, *Porphyromonas gingivali*s (*Pg*), and *S. sanguis/S. gordonii* was flood inoculated onto the surface of lysogeny broth (LB or blood LB) agar plates. Bacterial lawn was prepared by spread plate method with a volume of 100 μL of bacterial inoculum by using a micropipette (10–100 μL). Nutrition agar medium was placed in hot air oven for a period of 20 min to achieve suitable sterilization. The specimens with indicated concentrations of ZnO NPs (0.2, 0.4, 0.6, 0.8 μmol·L^−1^) with or without 100 µmol·L^−1^ of minocycline were placed on LB agar plates having bacterial strain and incubated 37 °C for 24 h. The diameter of each well is 6 mm and 10 μL of drug was dripped into it each time by using a micropipette. Zones of inhibition around the specimens were measured in millimeters using vernier caliper. The bacteria without Alb NPs were used as control and the culture medium without bacteria was considered as background.

#### 2.2.6. Cytotoxicity of the drug delivery system on gingival cell

Cell death/cell viability was assessed by a standard CCK-8 assay. The gingival cell was cultured in DMEM medium supplemented with 10% FBS at 37 °C in a humidified atmosphere with 5% CO_2_. The medium was renewed every 1–2 d. Subculture was performed every 3–4 d at a ratio of 1–3. The cells were harvested with 0.02% EDTA and 0.025% trypsin and rinsed. The resulting cell suspension was used in following experiments.

A gingival cell culture with a density of 1 × 10^4^ cells per well was cultured in a 100 μL volume of cell culture medium (DMEM) in a 96-well plate. The plate was incubated at 37 °C with 5% CO_2_ to obtain 70% confluency. It was treated with 10 μl of different concentration of Mino-ZnO@Alb NPs in DMSO starting from 0.2 to 1 μg/ml and incubated for 24 h. Subsequently, 10 μl of CCK-8 (1 mg/mL) was added to all the wells. After incubator for another 1.5 h, cell viability and cytotoxicity were evaluated by a microplate reader (SpectraMax M2, MDC, USA) at the absorbance of 450 nm.

#### 2.2.7. Determination of bioadhesive force of hydrogel

The bioadhesive force of hydrogel was determined using texture analyzer (CT3 100 g, Brookfield, Germany) on a special testing assembly with TA10 cylinder probe (12.7 mm D, 35 mm l) and maintains the temperature of 37 °C.

Briefly, rats were anesthetized with intramuscular injection of 10% chloral hydrate (3 mL/kg). After anesthetizing, the dorsal skin region of the animals was shaved to remove any hair present on the skin. Following this, 1 cm^2^ area and 0.5 cm full thickness incision was made on the dorsal skin using a sterilized surgical blade. The dermal site was used for the measurement of bioadhesive force. An excised rat skin was placed inside the bioadhesion assembly and on the bottom of the probe (1 cm diameter) hydrogel was fixed using double-sided tape. Test was run in the compression mode setting the target of 100 g and holds time of 15 s and the trigger load of 3 g. Test and return speed was kept 0.5 mm/s and the bioadhesive force was calculated by the following equation.

Bioadhesive force (N) = *W* (g·cm^−2^)× *G*/1000, where *W* is the weight required for the detachment of two skins in gram per square centimeter, and *G* is the acceleration of gravity.

#### 2.2.8. Determination of drug release from the pH-responsive hydrogel

Minocycline released from the hydrogel was investigated *in vitro* by dialysis method. The hydrogel (1 mL) were dispersed in 5 mL PBS in a dialysis membrane bag (MWCO = 3500), and the dialysis bag was immersed in 100 mL PBS with different pH value (pH 6.5 and 7.4) and gently shaken at 100 rpm at 37 °C. At predetermined sampling times, 0.5 mL aliquots were withdrawn and centrifuged at 14,000 rpm for 10 min. The amounts of minocycline in the supernatants were determined by UV spectrophotometer at the absorbance of 236 nm using linearly regressed standard curve. The removed fluid was instantly replaced with an equal amount of fresh dissolution medium. The amount of minocycline loaded into the nanoparticles was estimated by subtracting the amount of minocycline in the collected supernatant from the initial amount of minocycline in the loading solution using UV spectrophotometer.

#### 2.2.9. Establishment of periodontitis rat model

A total of 24 SD rats (male, 10 weeks, 300 ± 20 g) were housed in standard cages and allowed free movement and access to food and water during the whole experiment. Then the rats were randomly assigned into the following four groups: normal group, blank hydrogel control group, Mino-ZnO@Alb hydrogel treated group and Perio^®^ treated group.

Rats were anesthetized by chloral hydrate (10%, 0.3 mL/100g) and fixed on the plank in the supine position. To allow biofilm accumulation, the mandibular first molar of the rats received a 0.2 mm wire ligature knotted in the cervical one circle, which immersed into the gingival totally. The contralateral first molar was left unligated to be acted as a control. Under the circumstance of other experimental conditions be equal, the rats fed with 20% of sugar water instead of purified water. The probing pocket depth, bleeding index, and the clinical attachment loss were measured before and after treatment for two weeks as the important detection index to evaluate the periodontal disease *in vivo*.

#### 2.2.10. Histological analysis

At day 14 post implantations, the rats were euthanized (killed by CO_2_ inhalation) and then the mandibles were removed and hemisected. The ligatures were removed using a dental nipper. The tissue sections from the left side were fixed in 4% paraformaldehyde (PFA) for 48 h, washed with PBS, decalcified in 5% nitric acid in 0.1 M Tris solution (pH 7.0) for 72 h, washed with PBS and embedded in paraffin after series dehydration. For histological analysis, the sectioned tissues were stained with hematoxylin and eosin. The stained sections were observed using Nikon Eclipse TS 100 inverted routine microscope (Nikon, Japan).

#### 2.2.11. Statistical analysis

The results are expressed as mean ± standard deviation (SD, *χ*±*s*) and were analyzed by Student’s *t*-test for two groups and one-way ANOVA for multiple groups. The results were considered to be statistically significant at *p < .05 and **p < .01.

## 3. Results

### 3.1. Characterization of Mino-ZnO@Alb NPs

**Figure 1.**
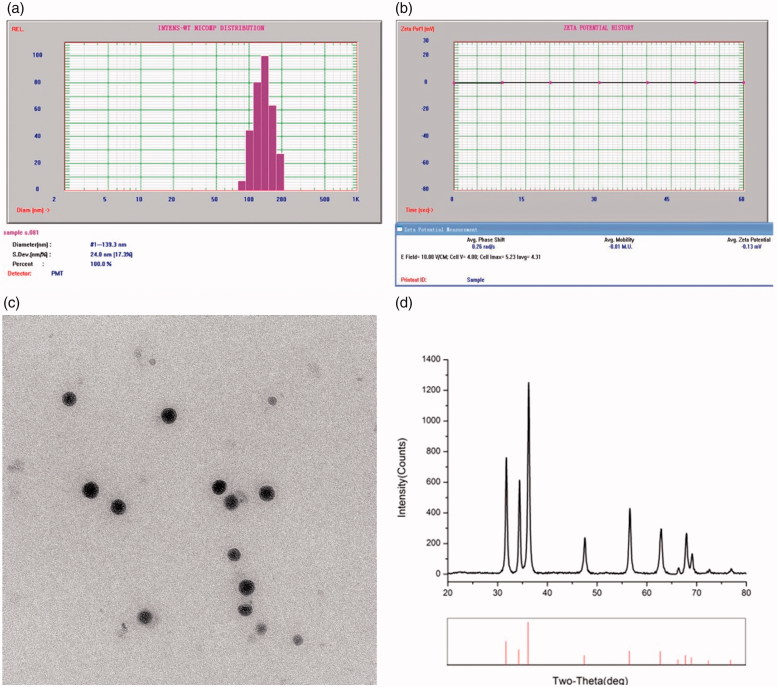
Characterization of Mino-ZnO@Alb NPs. (A) Particle size distribution of Mino-ZnO@Alb NPs. (B) Average Zeta potential of Mino-ZnO@Alb NPs. (C) The image of ZnO NPs scanned under transmission electron microscope. (D) XRD graph of ZnO nanoparticles.

The average diameter, particle size, and distribution of Mino-ZnO@Alb NPs were measured with NICOMP 380ZLS. The distribution of sphere diameters was shown in Figure 1(A). The image revealed that the average diameter was 133 nm and in the narrow size distribution. Figure 1(B) showed the average Zeta potential was −0.13 ev. The morphology of the micelles was observed by TEM. As depicted in Figure 1(C), the nanoparticles exhibited a subsphaeroidal shape and smooth surface. The size calculated from TEM was comparably smaller than the size observed by DLS. The difference in the mean size could be due to the fact that DLS size corresponds to the size of particles in solution, whereas the electron micrographs show the size of particles in a dried state. The particle diameters ranged from 100 to 150 nm, as evidenced by Zata-dimeter measurement. The formation of Mino-ZnO@Alb NPs was investigated by X-ray diffraction (XRD), which was depicted in Figure 1(D). All XRD diffraction peaks of ZnO are shown in a good agreement with hexagonal structure of zincite phase reported in JCPDS file card No. 05-0664.

### 3.2. EE and LD in Alb NPs

The ratio of minocycline and ZnO effects on basic character of nanoparticals were investigated (Supplementary Table 1). With the decreasing of drug–albumin ratio, EE values showed a clear rising trend from 81.04% to 89.99% (without ZnO NPs) and 92.21% to 99.00% (with ZnO NPs). When the drug–albumin ratio was 1:5, LD value reached the peak as 10.45% (without ZnO NPs) and 15.76% (with ZnO NPs). However, the ratio of drug/albumin and the addition of ZnO NPs had no apparent statistical difference on the particle sizes. With the addition of ZnO NPs, the particle sizes verified in a narrow range. It was indicated that the presence of ZnO NPs was conducive to the stability of Alb nanoparticles and increase EE values. This is probably because ZnO NPs could load the antibacterial drug based on the coordination bonding formation of ZnO-Alb and ZnO-drug molecules, respectively, thus increase the EE and LD to avoid release under the physiological condition caused by the dissolution of Alb NPs.

### 3.3. Effects of the formulation on the bioadhesive force of hydrogel

**Figure 2.**
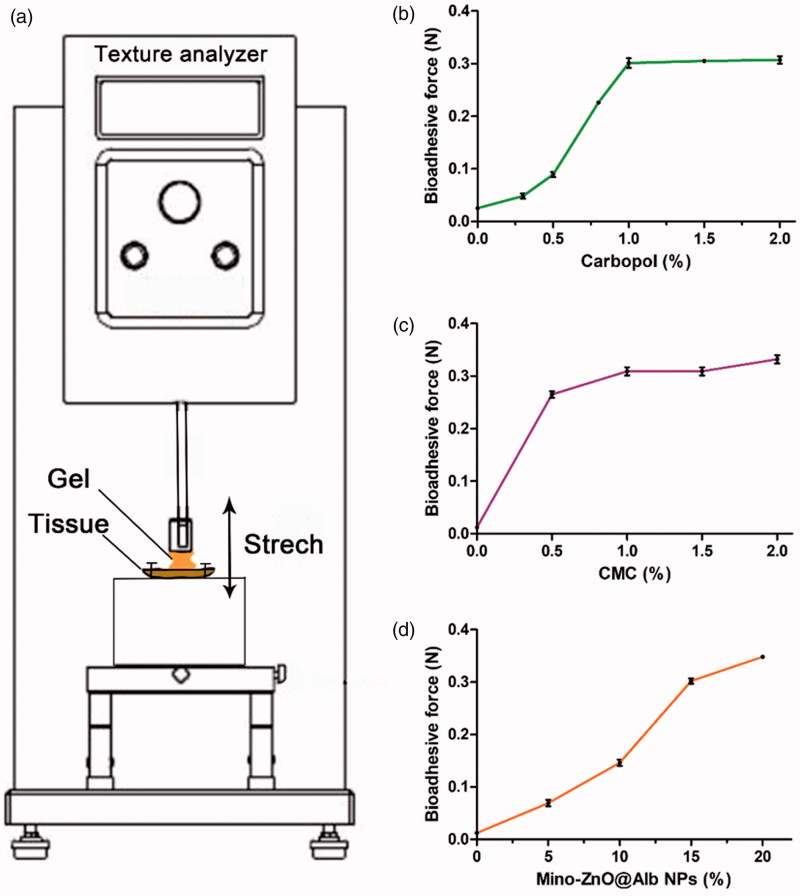
Effects of the formulation on the bioadhesive force of hydrogel. (A) The adhensive force measured by the Texture analyzer. (B) Effects of Carbopol concentration on the bioadhesive force of the hydrogel containing 1% CMC. (C) Effects of CMC concentration on the bioadhesive force of the hydrogel containing 1% Carbopol. (D) Effects of Mino-ZnO@Alb NPs concentration on the bioadhesive force of the hydrogel containing 1% Carbopol.

Due to the excellent biological compatibility with the tissues, carbopol 940^®^ was served as the gel stroma to prepare Mino-ZnO@Alb hydrogel. The relation of the amount of metallic oxide NPs, carboxymethyl cellulose (CMC) and carbopol 940^®^ with adhesive force of the hydrogel were investigated (Figure 2).

It was illustrated that Carbopol abruptly increased the bioadhesive force as the concentration increased from 0.3% to 1.0%. But the bioadhesive forces slightly changed when the concentration exceeded 1% (Figure 2(B)). CMC also significantly increased the bioadhesive force of the hydrogel. In the presence of CMC, the bioadhesive force of hydrogel was increased from 0.275 to 0.325 N (Figure 2(C)). It was noteworthy that Mino-ZnO@Alb NPs efficiently increased the bioadhesive force. In the absence of CMC, 20% Mino-ZnO@Alb NPs strengthened the bioadhesive forces as much as 25 fold. The results have shown that the bioadhesive force of hydrogel was enhanced by adding Mino-ZnO@Alb NPs or CMC. When the content of Mino-ZnO @Alb NPs is further increased, the cytotoxicity is also improved. From the findings, 1% of Carbomer 940, 20% of Mino-ZnO@Alb NPs were the optimum matrix of Mino-ZnO@Alb hydrogel.

### 3.4. Antibacterial activity screening

**Figure 3.**
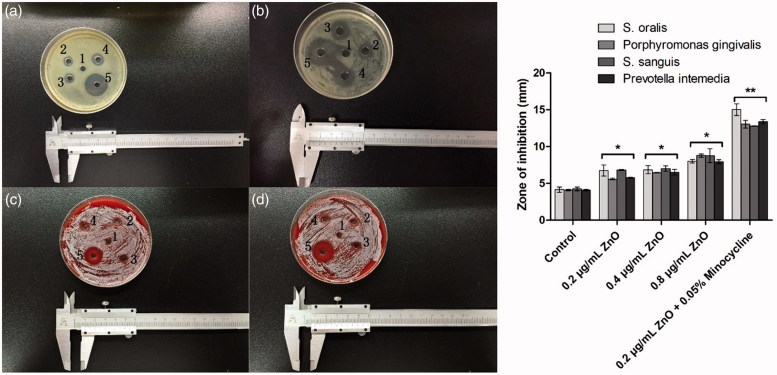
The image of the zones of growth inhibition of *S. oralis*, *Porphyromonas gingivali*s, *S. sanguis*, and *Prevotella intemedia* under the nanoparticals. (A) *Prevotella intemedia*, (B) *S. oralis*, (C) *Porphyromonas gingivali*s, and (D) *S. sanguis.* Five wells of each plate are filled with specimens in the following order. (1) blank NPs as the control group, (2) 0.2 × 10^−3^ g·L^−1^ ZnO@Alb NPs, (3) 0.4 × 10^−3^ g·L^−1^ ZnO@Alb NPs, (4): 0.8 × 10^−3^ g·L^−1^ ZnO@Alb NPs, and (5) ZnO-Mino@Alb NPs (0.2 × 10^−3^ g·L^−1^ ZnO + 0.5 g/L minocycline). *p < .05 compared with the control group; **p < .01 compared with the control group.

The inhibition zone method was employed to evaluate the antibacterial activity of Mino-ZnO@Alb NPs (Figure 3). After 24 h cultivation, the inhibition zones to the four strains grow larger when ZnO was added from 0.2 to 0.8 mg/L in Alb NPs, but the combination of minocycline (500 mg/L) and ZnO (0.2 mg/L) in Alb nanoparticles presented prominent implications to the four bacteria strains. The inhibition zone diametres of Mino-ZnO@Alb NPs to *S. oralis*, *Porphyromonas gingivali*s, *S. sanguis,* and *Prevotella intemedia* were 15.10, 13.36, 12.98, and 14.01 mm, respectively, which showed remarkable synergistic effects of the orgainc and inorganic antibacterial agents. The results also indicate that the no resistant appeared to all of the drug formulations.

### 3.5. *In vitro* drug release

**Figure 4.**
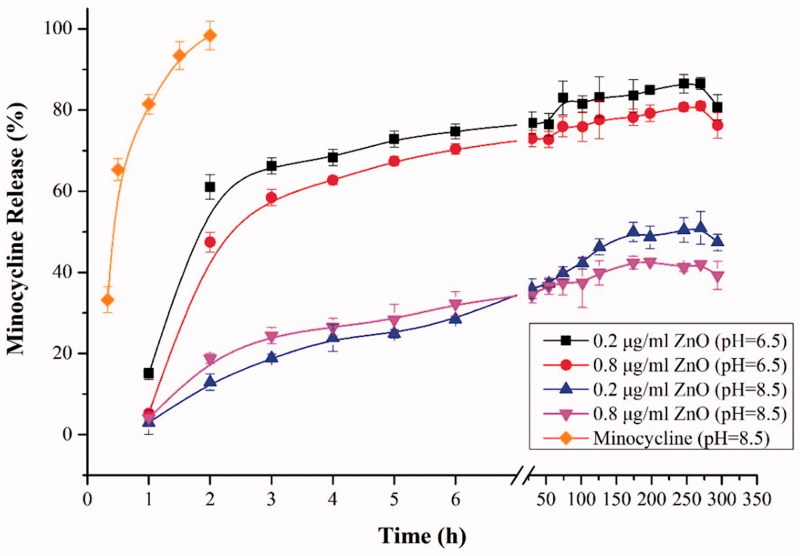
The pH-sensitive release profiles of minocycline from Mino-ZnO@Alb NPs hydrogels.

Drug release profiles of minocycline from the hydrogels as a function of pH value in the phosphate buffer system are depicted in Figure 4. *In vitro* release behavior of Mino–Zn@Alb NPs showed a fine pH-responsiveness. The release mount at pH 6.5 was close to 80%, while the cumulative release amount at pH 7.4 was less than 50% within 24 h and the release amount was close to 60% within 300 h (Figure 4). Different contents of ZnO in Mino-ZnO@Alb NPs showed similar release patterns differing only slightly in terms of cumulative drug release. It was found that free minocycline released rapidly and reached 100% cumulatively less than 2 h, while drug released from hydrogels increased steadily up to 120 h. In case of micelles, an initial fast release was observed within first 3 h (∼70%). At pH 6.5, 80% minocycline was released over a five-day period, whereas only about 40% minocycline was released in the same period at pH 8.5 which could be due to higher partitioning of minocycline at acidic condition. This could be helpful and add to the advantage of selective and targeted uptake in inflammatory cells where the pH is slightly acidic compared to normal physiological pH value. It could be suggested that the pH-sensitive behavior of micelles was a result of the combined effects of minocycline partitioning in acidic media and relaxation or swelling of nanoparticals in solution over time.

### 3.6. Effects of hydrogel on rat periodontal disease model

**Figure 5.**
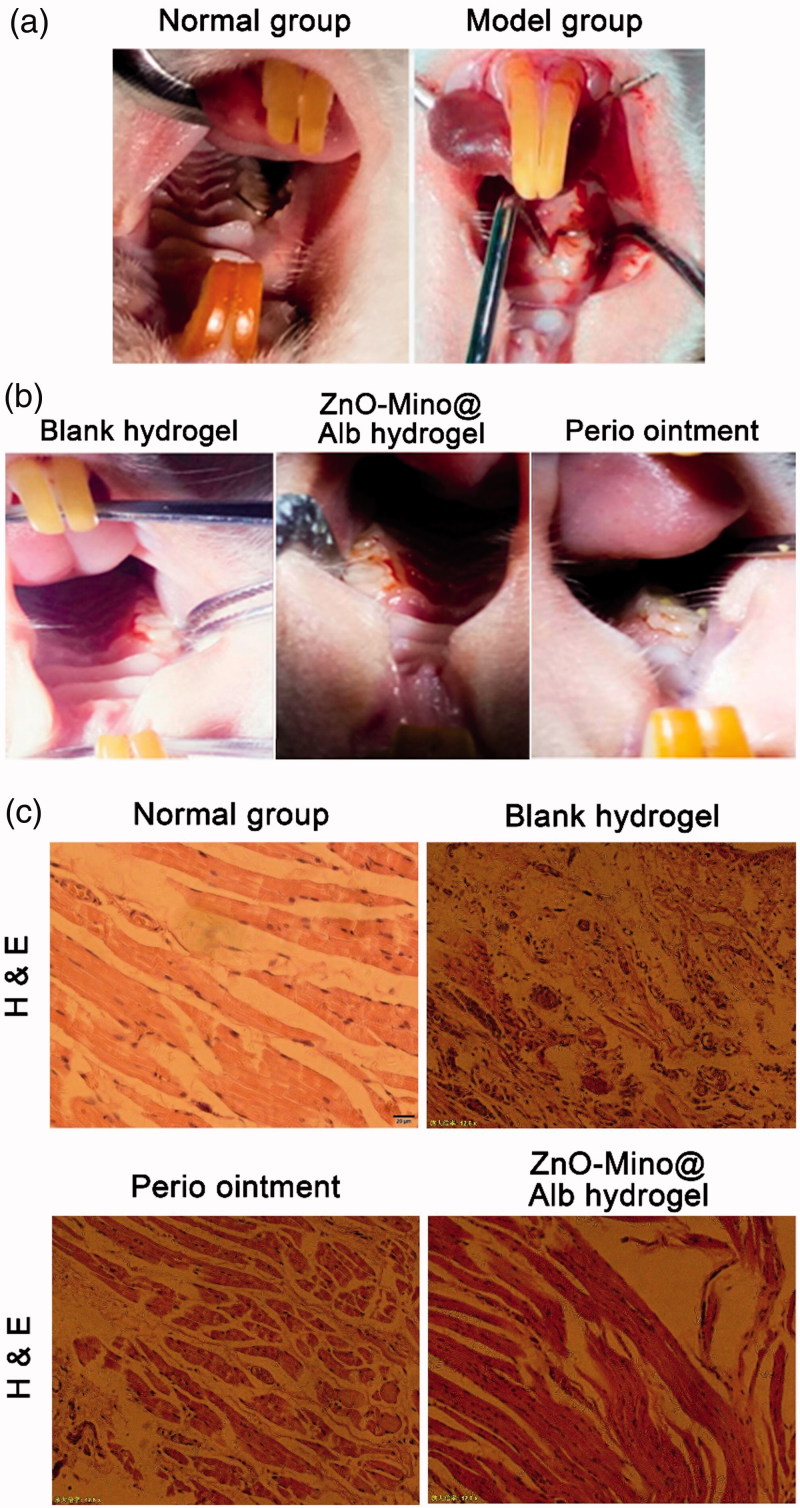
(A) Construction of the periodontitis model by ligating the first odontoprisis on the top jaw of rats. (B) Morphological of the periodontitis model in rats after treatment for two weeks. (C) Photos of H&E stained gingival tissue sections after two weeks of implantations of different drugs, magnification (200×).

Compared to the normal rat, it was obviously observed the distinct redness and swelling of the gingival near the first molar of the left side (Figure 5(A)). It illustrated that the rat periodontitis disease model has been established successfully. All sections exhibited clear signs of inflammation within the rat periodontitis disease model.

The morphological of the periodontitis model in rats after treatment for two weeks were showed in Figure 5(B). It was illustrated that the gingival redness and swelling disappeared obviously in Mino-ZnO@Alb hydrogel treated group. The probing pocket depth, bleeding index, and the clinical attachment loss were measured as the most important detection index for evaluating the effects of drugs on periodontitis disease *in vivo* (Supplementary Table 4). Compared to the model group, the detection index in Mino-ZnO@Alb hydrogel treated group was significant decreased (**p < .01), which is equal or better than Perio^®^ group. The results indicated that the accessorial effect of Mino-ZnO@Alb hydrogel on periodontitis is more apparent.

From Figure 5(C), the gingival tissue histology showed that the inflammatory cell infiltrate was usually clearly defined and often adjacent to areas of dense collagen bundles in the normal group. The significant differences in the size or density of the inflammatory cell infiltrate in the connective tissue were noted in the periodontitis disease model group. No inflammatory round cell infiltrates in the connective tissue were observed between the Perio^®^ and nanohydrogel treated groups due to the antibacterial effects. All defects containing the implanted hydrogel had been repaired by tissue promotion from the gingiva; the biomaterial particles had served as tissue conductive scaffolds. Moreover, staining was more intense than in surrounding tissue after thionine staining.

### 3.7. Cytotoxicity evaluation of gingival cells against Mino-ZnO@Alb NPs

To evaluate the toxicity of the antibacterial nanoparticals, the CCK-8 assay was employed on the gingival cell after treated for 24 h incubation. The results exhibited that the cell viability were above 85% and slightly decreased in a dose-dependent manner when ZnO contents increased from 0.2 to 0.8 mg/L.

**Figure 6.**
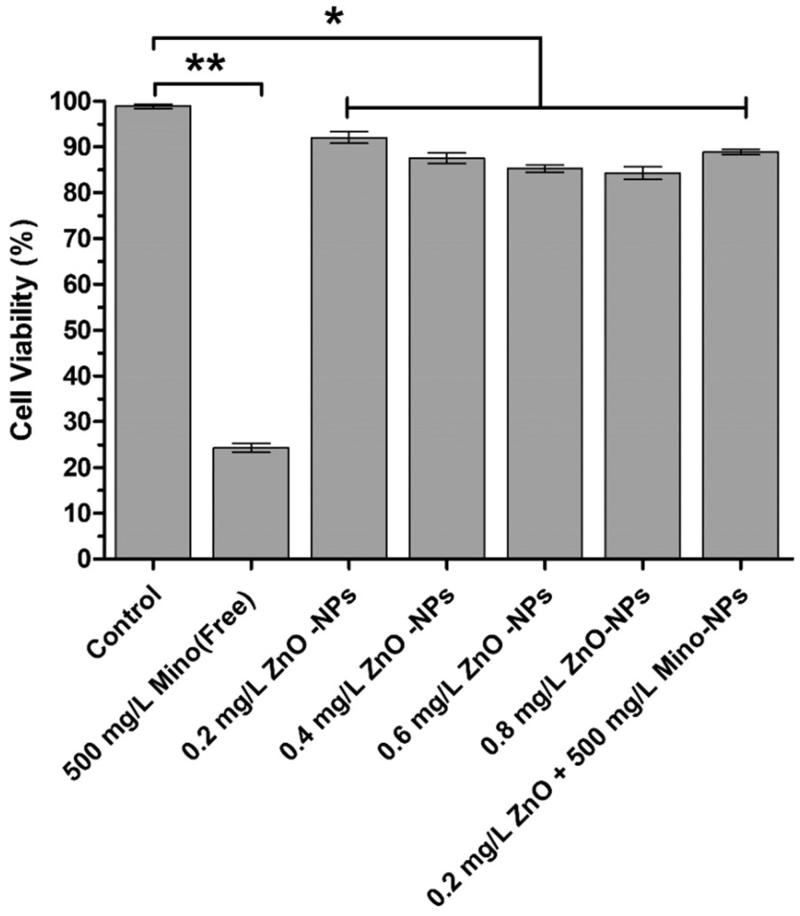
*In vitro* cytotoxicity of ZnO@Alb NPs and Mino-ZnO@Alb NPs against Gingival cells for 24 h. Each point represents mean ± SD (*n* = 3). *p < .05 compared with the control group, **p < .01 compared with the control group.

Except for 500 mg/L minocycline treated group, other groups did not show any toxic activity in relation to the gingival cells and did not exhibit any decrease in survival of the cells below the threshold of 70%. Mino-ZnO@Alb NPs exhibited lower cytotoxicity than Mino groups with more than 80% of cells were viable after 24 h of incubation (Figure 6). Cytotoxicity studies indicated that the association of ZnO and minocycline had prior synergistic effect than ZnO exclusive administration. However, when blank Alb nanoparticles were used, no significant difference was observed among the Mino-loaded NPs. It can be suggested that this enhanced cytotoxicity probably resulted from the combination of enhanced synergistic effect of ZnO and Minocycline.

## 4. Discussion

The antimicrobial chemotherapy, especially tetracycline, adjunctively performed with SRP were applied for moderate to severe periodontitis. Minocycline, the representative kind of tetracyclines, is active against important periodontal pathogens such as *A. actinomycetemcomitans*; it also has anti-collagenase properties to reduce tissue destruction and bone resorption (Jusko et al., 2016; Olsen et al., 2017; Pokrowiecki et al., 2017). However, the commercial minocycline oniment (Perio^®^, 2% dosage of minocycline ointment) inevitable enhanced the adverse effects including the retardation of bone growth, photosensitivity, permanent discoloration of developing teeth, teratogenesis, as well as hepatic and renal toxicity in susceptible individuals (Abbas et al., 2016). Besides, the generation of bacterial resistance is another major challenge that extremely restricts the benefits of minocycline in controlling infections. To circumvent this problem, the combination of antibacterial agents has become an actively explored strategy.

Recently, metal oxides (i.e. CuO, TiO_2_, ZnO, Al_2_O_3_, SiO_2_, Fe_2_O_3,_ and CeO_2_) are extensively investigated in the field of inorganic antibacterials to avoid multidrug resistance. It was found that their activities are easily tunable by control over their particle size, morphology, and crystal defects through appropriate synthesis methods (Nakao et al., 2011; Khan et al., 2016). Znic oxide (ZnO), which was approved as the safe material by FDA (21 CFR 182.8991) (Dizaj et al., 2014), was selected for its moderate antibacterial activity against both Gram-positive and negative bacteria. It was reported the gelatin nanofibers loaded with zinc oxide nanoparticles (ZnO NPs) and cefazolin were fabricated to maximize the antibacterial efficiency and reduce the chances of resistance development in the microbes (Vimbela et al., 2017). However, clinical development of nanoparticles is challenging because of their limitations in physicochemical properties, such as low drug loading efficiency and poor circulation stability. The novel strategy of the nanostructured inorganic metal oxide and antibacterial drug carried in biocompatible and biodegradable delivery systems has been proposed for the more efficient and encouraging approaches devoid of the undesirable effects (Chen et al., 2014).

Albumin is the most abundant protein in plasma (35 ∼ 50 g·L^−1^ human serum), which was widely used as the biodegradable carrier for drug delivery (Soppimath et al., 2001; Chuang et al., 2002; Wu et al., 2009). The albumin-based drug delivery system can increase the ratio of drug concentration in the inflammation tissue to the normal tissue (Maranhão et al., 2017). Taking together, the low immunogenicity, nontoxicity, biodegradability, and preferential uptake in inflammation make serum albumin an ideal candidate carrier for anti-inflammatory drugs delivery (Karimi et al., 2016; Maranhão et al., 2017). Zn-loaded bovine serum albumin nanoparticles (Zn-BSA NPs) were reported as the carriers for pH-responsive anticancer drug delivery (Li et al., 2012). The use of the stimuli-responsive drug delivery systems offers an interesting opportunity for drug and gene delivery where the delivery system becomes an active participant, rather than a passive vehicle, in the optimization of therapy (Ganta et al., 2008). The pH-responsive system is of special interest for its importance in chemotherapy of inflammation. Antibiotics incorporated into sustained-release vehicles at lower doses than in systemic administration have been developed and clinically applied which is known as a local drug delivery system (LDDS) (Matesanz-Pérez et al., 2013). A variety of hydrogels, with slow-release effect, have been utilized as carrier materials for drug delivery, which can offer structural integrity and cellular organization performing as bioadhesive drug depots, deliver bioactive substances and own exclusive swelling properties and structure (Singh et al., 2013; Yang et al., 2017).

In this study, the synergistic effect of Minocycline and ZnO NPs for antimicrobial in a biocompatible and biodegradable carrier was explored. The effects of lessening the cytotoxic drug dosage with more efficient partial objective of the drug, reducing the associated side effects with multi advantages were highlighted as the novel aspect of this study.

From the pharmaceutical viewpoint, the ultimate important parameter to the nano-structured drug delivery system is the EE and LD. In general, the higher EE and LD values of nanoparticals mean more effective drugs that can be used in the drug delivery system. It was indicated that the presence of ZnO NPs was conducive to the stability of Alb nanoparticles and increase EE values. This is probably because ZnO NPs could load the antibacterial drug based on the coordination bonding formation of ZnO–Alb and ZnO–drug molecules, respectively, thus increase the EE and LD to avoid release under the physiological condition caused by the dissolution of Alb NPs.

## 5. Conclusion

In conclusion, we developed a Mino-ZnO@Alb nanohydrogel that exhibited unprecedented multifunctional properties, including pH-responsiveness, wide antimicrobial spectrum, sustained release, tissue-repairing, and adhesive properties. Compare to Perio^®^, Mino-ZnO@Alb hydrogel could be a promising product as it can increase bioavailability, minimize the dosage of the minocycline, reduce the side effects and improve patient compliance. The combination of metal oxide nanoparticals might be a right and suitable candidate for controlled drug delivery via bioadhesive hydrogel. The procedure can increase stability and is an example of specialized nanocarriers for improved drug delivery.

Further studies are needed to investigate these formulations for its performance in pharmacokinetics, *in vivo* studies on higher animals, and controlled clinical studies on human beings able to bring the product into the market.

## Supplementary Material

2019-1-9-revised_Supplementary_Information.pdf
