# Supplementary material for: Hydrogel containing minocycline and zinc oxide-loaded serum albumin nanopartical for periodontitis application: preparation, characterization and evaluation
Source: Drug Deliv. 2019 Mar 1;26(1):179–87. doi: 10.1080/10717544.2019.1571121 (PMC6407595; doi:10.1080/10717544.2019.1571121)
Supplement: 2019-1-9-revised_Supplementary_Information.pdf [file IDRD_A_1571121_SM0894.pdf]

Electronic Supplementary Information (ESI)

Hydrogel containing minocycline and zinc oxide loaded serum albumin nanopartical for periodontitis application: preparation, characterization and evaluation

Jie Mou[a,b,*], Zongxiang Liu[c,#], Jie Liu[a, #], Jianwu Lu[b], Dongsheng Pei [d,*]

1 Strategy for the pH-responsive release system

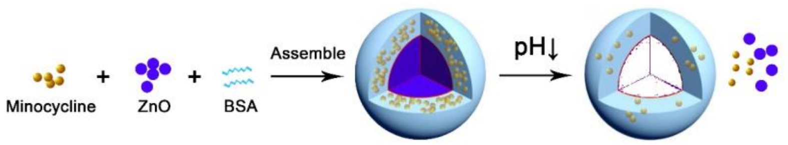

Scheme 1. The strategy for the pH-responsive shell-core structured antibacterial drug delivery system.

2 The morphology of ZnO NPs

The transmission electron microscope of ZnO NPs was added as S-Fig 2. It has demonstrated the ZnO NPs are inclined to agglomerate and exhibit hexagonal structure of zincite phase which is in consist with XRD result. Compared the two TEM images of Fig 1C and S-Fig 2, it could be found the significant differences on the morphology of ZnO NPs and Mino-ZnO@Alb NPs. Mino-ZnO@Alb NPs exhibit subsphaeroidal shape with smooth surface and uniformly dispersed due to surface charge after surface modified by HSA.

批注 [m1]: Review1-3

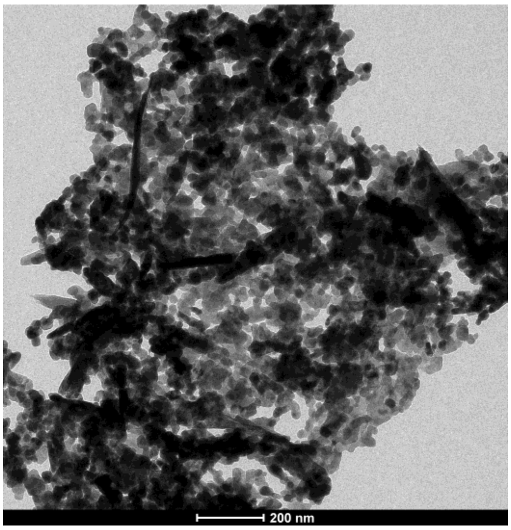

S-Fig 1 The image of ZnO NPs scanned under transmission electron microscope.

3 Optimization of encapsulation efficiency and drug-loading content in Alb NPs

The ratio of minocycline and ZnO effects on basic character of nanoparticals were investigated in S-Table1.

S-Table 1. The basic characterizations of nanoparticles

Drug ratio[a] (minocycline : albumin)		Particle Size (nm ± SD)	Drug Loading (% ± SD)	Encapsulation Efficiency (% ± SD)
without ZnO NPs	1:3	142 ± 6	8.08 ± 0.10	81.04 ± 1.36
	1:5	152 ± 4	10.45 ± 0.08	87.73 ± 2.78
	1:7	133 ± 6	9.40 ± 0.05	88.95 ± 2.32
	1:9	149 ± 11	8.20 ± 0.11	89.99 ± 2.45
with ZnO NPs	1:3	140 ± 7	13.32 ± 0.16	92.21 ± 2.56
	1:5	146 ± 9	15.76 ± 0.13	95.56 ± 2.25
	1:7	140 ± 4	11.96 ± 0.08	98.24 ± 1.64
	1:9	139 ± 5	9.86 ± 0.06	99.00 ± 1.07

[a] Drug ratio refers to minocycline to albumin (mg: mg)

To optimize the technology process, the parameters including the stirring speed, temperature, pH value and crosslinking agent content were investigated. It was found

that the partials size of Mino-ZnO@Alb NPs were more influenced by temperature than the stirring speed and time (S-Table 2).

S-Table 2 Influence of process parameters on particles size and drug loading

Run	Stirring speed (rpm)	Crosslinking ratio[b]	Temperature (° C)	Drug Loading (% ± SD)	Particle Size (nm ± SD)
1	400	1	25	9.08 ± 0.10	222 ± 7
2	400	3	25	10.45 ± 0.08	246 ± 5
3	400	6	25	9.40 ± 0.05	280 ± 6
4	400	1	4	6.20 ± 0.11	214 ± 4
5	400	3	4	6.60 ± 0.11	249 ± 7
6	400	6	4	6.70 ± 0.11	285 ± 12
7	600	1	25	13.32 ± 0.16	146 ± 5
8	600	3	25	15.76 ± 0.13	153 ± 5
9	600	6	25	14.96 ± 0.08	182 ± 8

[b] Crosslinking ratio refers to 25% glutaraldehyde and Alb (mg: mg)

The temperature maybe firstly related to albumin fluidity, thus had an effect on the drug encapsulation. The loading amount of minocycline was increased with pH values ranging from 5 to 10 (S-Table 3). When pH value increased above 7, the loading amounts of minocycline sharply increased, suggesting a strong affinity between minocycline and ZnO NPs at basic conditions.

S-Table 3 Effects of pH value on drug loading

pH	5	6	7	8	9	10
Drug Loading (% ± SD)	6.74 ± 0.12	9.86 ± 0.06	13.32 ± 0.16	14.76 ± 0.13	14.86 ± 0.09	15.13 ± 0.20

4 Stability of drug delivery system

Because nanoparticles tend to agglomerate, the stability of the drug delivery system was evaluated by the release profiles of minocycline from Mino-ZnO@Alb NPs hydrogel. The drug release level from the nanohydrogels were measured between the

samples prepared one month ago and twelve months ago. There was no significant difference in the drug release profiles which indicating the drug delivery system is very stable (S-Fig 2).

批注 [m2]: Reviewer 2-5

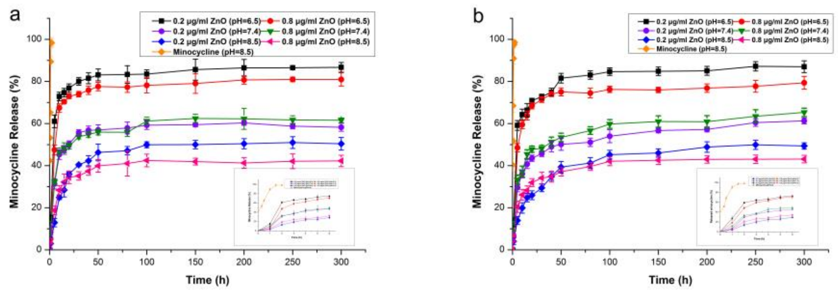

S Fig 2 The release profiles of minocycline from Mino-ZnO@Alb NPs hydrogels. (a) The sample prepared one month ago. (b) The sample prepared twelve months ago.

5 Effects of minocycline contents of Mino-ZnO@Alb NPs on cytotoxicity

To evaluate the effects of minocycline contents of Mino-ZnO@Alb NPs on cytotoxicity, the CCK-8 assay was performed on gingival cells after 24 hours of incubation. The results showed that the contents of minocycline in Mino-ZnO@Alb NPs increased from 500 mg/L to 700 mg/L, the cell viability was above 80%. When the Mino-ZnO@Alb NPs content exceeds 800 mg/L, the cell viability was sharply decreased. It can be considered that this enhanced cytotoxicity may be due to an increase of minocycline contents of Mino-ZnO@Alb NPs.

批注 [m3]: Reviewer 1-5

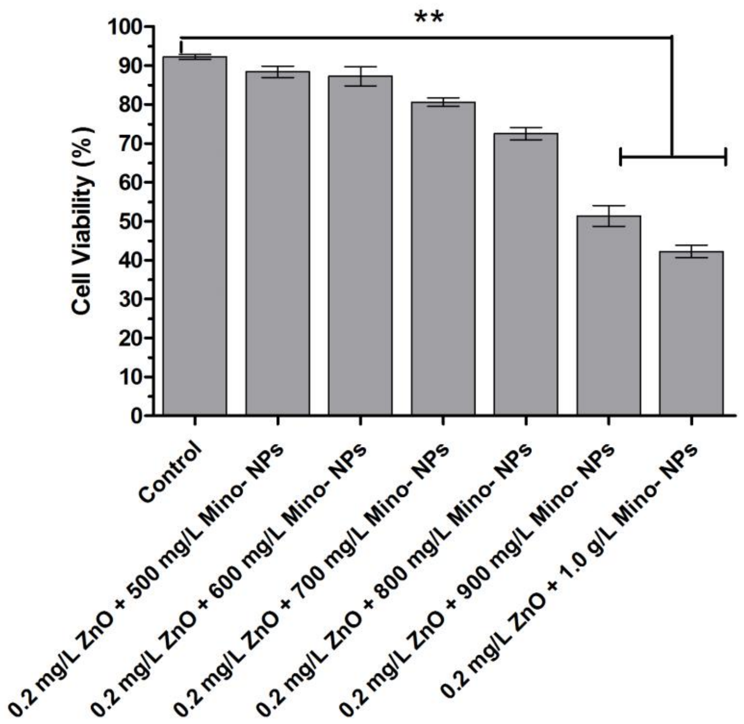

S-Figure 3 In vitro cytotoxicity of minocycline contents of Mino-ZnO@Alb NPs against Gingival cells for 24 h. Each point represents mean ± SD (n=3). ***p* < 0.01 compared with the control group.

6 Effects of hydrogel on rat periodontal disease model

The probing pocket depth, bleeding index and the clinical attachment loss were measured as the most important detection index for evaluating the effects of drugs in periodontitis disease in *vivo* (S-Table 4). Compared to the model group, the detection index in Mino-ZnO@Alb hydrogel treated group were significant decreased (*p*** < 0.01), which is equal or better than Perio® group. The results indicated that the accessorial effect of Mino-ZnO@Alb hydrogel on periodontitis is more apparent.

S-Table 4 Evaluation index of rats with periodontitis after treatment for two weeks (n=6, x±s)

Treatment groups	Depth of periodontal pocket (PD, mm)	Bleeding index (BI)	Periodontal attachment loss (AL)
Model group	1.65±0.16	4.47±0.08	3.78±0.24
Blank hydrogel	1.57±0.23	4.14±0.26	3.46±0.32
Perio® ointment	0.98±0.12**##	2.09±0.16**##	1.47±0.22**##

ZnO-Mino@Alb hydrogel	0.74±0.21**##	1.22±0.11**##	1.13±0.18**##

**$p < 0.01$, compared with the model group; ##$p < 0.01$, compared with the blank hydrogel group

7 Possible kinetic models in Mino-ZnO@Alb NPs hydrogel

Minocycline released kinetics from Mino-ZnO@Alb NPs (containing 0.2 or 0.8 mg/ml ZnO) and minocycline PBS solution were analyzed using the release kinetic models including zero-order equation, frst-order equation, Higuchi model, Ritger-peppas model and Weibull model. The calculated determination coefficients (R^2) were summarized in S-Table 5. Compared the R^2 value, it was found that the first-order equation could more suitable for the minocycline released curves at different pH values.

批注 [m4]: Reviewer 2-4

S-Table 5 Analysis kinetic models of Minocycline released rate from Mino-ZnO@Alb NPs hydrogel

批注 [l5]: Reviewer:2-4

Formulation (ZnO, μg/ml)	Model name	Model equation	R^2 pH 6.5	pH 7.0	pH 8.5
Mino-ZnO@Alb NPS(0.2 mg/ml ZnO)	Zero-order	$Q = \alpha + \beta t$	0.7472	0.7293	0.6334
	First-order	$In(1-Q) = \alpha + \beta t$	0.9504	0.9583	0.9491
	Higuchi	$Q = \alpha + \beta t_{1/2}$	0.8828	0.8321	0.8265
	Ritger-peppas	$In\ Q = \alpha + \beta ln\ t$	0.7988	0.7329	0.9357
	Weibull	$In(-ln(1 - Q)) = ln\ \alpha + \beta lnt$	0.8201	0.8902	0.9798
Mino-ZnO@Alb NPS (0.8 mg/ml ZnO)	Zero-order	$Q = \alpha + \beta t$	0.2745	0.2041	0.4511
	First-order	$In(1-Q) = \alpha + \beta t$	0.9474	0.9413	0.9509
	Higuchi	$Q = \alpha + \beta t_{1/2}$	0.4625	0.4532	0.6523
	Ritger-peppas	$In\ Q = \alpha + \beta ln\ t$	0.7612	0.7832	0.8761
	Weibull	$In(-ln(1 - Q)) = ln\ \alpha + \beta lnt$	0.9366	0.9104	0.9181

S-Table 6 Analysis of Kinetic models in minocycline released rate from minocycline in phosphate buffer solution

Formulation	Model name	Model equation	R^2（pH 8.5）
Minocycline solution	Zero-order	$Q = \alpha + \beta t$	0.7732
	First-order	$In(1-Q) = \alpha + \beta t$	0.9788
	Higuchi	$Q = \alpha + \beta t_{1/2}$	0.9519
	Ritger-peppas	$In\ Q = \alpha + \beta ln\ t$	0.9798
	Weibull	$In(-ln(1 - Q)) = ln\ \alpha + \beta lnt$	0.9560
